# Utilizing IoT Sensors and Spatial Data Mining for Analysis of Urban Space Actors’ Behavior in University Campus Space Design

**DOI:** 10.3390/s25051393

**Published:** 2025-02-25

**Authors:** Krzysztof Koszewski, Robert Olszewski, Piotr Pałka, Renata Walczak, Przemysław Korpas, Karolina Dąbrowska-Żółtak, Michał Wyszomirski, Olga Czeranowska-Panufnik, Andrzej Manujło, Urszula Szczepankowska-Bednarek, Joanna Kuźmicz-Kubiś, Anna Szalwa, Krzysztof Ejsmont, Paweł Czernic

**Affiliations:** 1 Faculty of Architecture, Warsaw University of Technology, ul. Koszykowa 55, 00-659 Warsaw, Poland; karolina.dabrowska@pw.edu.pl; 2 Faculty of Geodesy and Cartography, Warsaw University of Technology, Plac Politechniki 1, 00-651 Warsaw, Poland; robert.olszewski@pw.edu.pl (R.O.); michal.wyszomirski@pw.edu.pl (M.W.); 3 Faculty of Electronics and Information Technology, Warsaw University of Technology, ul. Nowowiejska 15/19, 00-665 Warsaw, Poland; piotr.palka@pw.edu.pl (P.P.); przemyslaw.korpas@pw.edu.pl (P.K.); 4 Faculty of Civil Engineering, Mechanics and Petrochemistry, Warsaw University of Technology, ul. Łukasiewicza 17, 09-400 Plock, Poland; renata.walczak@pw.edu.pl; 5 Doctoral School, Warsaw University of Technology, ul. Rektorska 4, 00-614 Warsaw, Poland; olga.czeranowska-panufnik.dokt@pw.edu.pl (O.C.-P.); anna.szalwa.dokt@pw.edu.pl (A.S.); pawel.czernic.dokt@pw.edu.pl (P.C.); 6 Faculty of Power and Aeronautical Engineering, Warsaw University of Technology, Nowowiejska 24, 00-665 Warsaw, Poland; andrzej.manujlo@pw.edu.pl; 7 Center for Innovation, Warsaw University of Technology, ul. Rektorska 4, 00-614 Warsaw, Poland; urszula.bednarek@pw.edu.pl; 8 University Social Responsibility Office, Warsaw University of Technology, ul. Mochnackiego 10, 02-042 Warsaw, Poland; joanna.kuzmicz@pw.edu.pl; 9 Faculty of Mechanical and Industrial Engineering, Warsaw University of Technology, ul. Narbutta 85, 02-524 Warsaw, Poland; krzysztof.ejsmont@pw.edu.pl

**Keywords:** IoT networks, data management and analytics, smart cities, urban design, data-driven design, spatial data mining

## Abstract

This paper discusses the use of IoT sensor networks and spatial data mining methods to support the design process in the revitalization of the university campus of the Warsaw University of Technology (WUT) in the spirit of universal design. The aim of the research was to develop a methodology for the use of IoT and edge computing for the acquisition of spatial knowledge based on spatial big data, as well as for the development of an open (geo)information society that shares the responsibility for the process of shaping the spaces of smart cities. The purpose of the article is to verify the hypothesis on whether it is possible to obtain spatial–temporal quantitative data that are useful in the process of designing the space of a university campus using low-cost Internet of Things sensors, i.e., already existing networks of CCTV cameras supported by simple installed beam-crossing sensors. The methodological approach proposed in the article combines two main areas—the use of IT technologies (IoT, big data, spatial data mining) and data-driven design based on analysis of urban space actors’ behavior for participatory revitalization of a university campus. The research method applied involves placing a network of locally communicating heterogeneous IoT sensors in the space of a campus. These sensors collect data on the behavior of urban space actors: people and vehicles. The data collected and the knowledge gained from its analysis are used to discuss the shape of the campus space. The testbed of the developed methodology was the central campus of the WUT (Warsaw University of Technology), which made it possible to analyze the time-varying use of the selected campus spaces and to identify the premises for the revitalization project in accordance with contemporary trends in the design of the space of HEIs (higher education institutions), as well as the needs of the academic community and the residents of the capital. The results are used not only to optimize the process of redesigning the WUT campus, but also to support the process of discussion and activation of the community in the development of deliberative democracy and participatory shaping of space in general.

## 1. Introduction

The development of smart cities in the 21st century is stimulated not only by technological factors, but also by the greater involvement of the local community and the development of an open information society. According to BSI PAS 180:2014, “Smart cities is a term used to describe the effective integration of physical, digital, and social systems in urban spaces to provide a sustainable and prosperous living environment for citizens” [1]. The essence of this approach is the use of modern technology, IoT sensors, and spatial data mining methods to acquire spatial knowledge. Public participation that utilizes modern IT technologies, mobile devices such as smartphones, GNSS satellite geolocation, and diverse IoT sensors makes it possible not only to collect residents’ opinions, but also to stimulate their activity and make them jointly responsible for shaping urban space.

Efforts to design and implement solutions at the architectural and urban scale must be optimized in the context of limited access to resources [2]. This means more than design that minimizes the environmental impact and increases resilience in the face of adverse climate change. It also calls for a need to shape urban spaces to maximize the possibility of comfortable use by the largest possible group of people at a reasonable cost, applying relevant design strategies (universal design, compact city, incorporating brownfield sites, etc.) [3]. Such an approach requires technological support, because it relies largely on making multi-criteria decisions with a high degree of complexity [4,5]. Advanced spatial analysis techniques based on data collected by IoT sensors enable an empirically founded description of the use of space affected by architectural and urban design [6]. In order to minimize energy and resource consumption, it is important to also take into account the optimization of the data collection process itself, utilizing the widest possible range of already existing IoT sensor networks, like CCTV, thus minimizing infrastructure investments, while at the same time emphasizing the stability and security of such a network.

The way in which the physical space of a city is shaped, i.e., its morphology, has a significant impact on the movement characteristics of its users [7] and, consequently, on the efficiency of the use of a specific space, including ensuring the comfort of its users. Research in this area shows how relatively small changes in the designed layout of streets or the location of buildings can affect the intensity of pedestrian and vehicular traffic, both in typical urban spaces as well as in specific ones, such as university campuses [8]. Any flow analysis requires validated and sufficiently precise input data. Such data are constantly being collected by the numerous IoT sensor networks installed in cities [9]. Appropriate acquisition, processing, interpretation, and implementation of the results in the process of architectural and urban design is an important research topic and this paper focuses on its selected aspects.

The issue of adopting smart city solutions can be analyzed on a smaller scale using the example of university campuses. Due to the nature of technical universities, the use of CCTV cameras and IoT sensor networks on these campuses can be a kind of urban Living Lab, serving as an experimental implementation of the smart city idea. Universality in the design approach can also be associated with flexibility in the use of designed spaces. The need for flexible and scalable solutions in the shaping of urban spaces results from both post-pandemic conclusions and the specifics of the design problem. In this case it is the university campus, where places of informal integration are of considerable importance. The proper design of such spaces also requires a diligent study of user behavior.

According to its praxeological definition, designing is the conceptual preparation of a relevant change [10]. As with any change affecting a large number of users, it is therefore the result of negotiations and an area of confrontation of expectations. In this context, thorough analyses based on empirical data from IoT sensor networks, including image-based data, are of significant value in supporting the argument for discussion, social acceptance, and adoption of the proposed solutions [11]. They significantly support the traditional approach, in which the primary design premise was the experience of the designer and the analysis of analogous (but nonetheless different) cases.

## 2. Purpose of the Research

A rational design of a common open space at a campus requires the development of a concept with consideration of the needs of the local community and its habits, reflected in the usage patterns of this space. The general purpose of the research described in this paper was to study the impact of the application of IoT technology as a data driver for architectural and urban design, aiming to improve the quality of the space of the Central Campus of the Warsaw University of Technology. The research team aimed in particular to lay foundations for creating relatively low-cost analysis methods for the behavior of urban space actors, resulting in a set of analyses informing future design decisions. Thus, the goal of the research carried out using data collected by IoT sensors is primarily to automate and objectify the process of analyzing how space is used and to determine the spatial and temporal variation in the use of that space. The proposed research question is whether it is possible to obtain spatial–temporal quantitative data allowing for behavior analysis of urban space actors, resulting in a set of conclusions informing future design decisions and maintaining low costs for the proposed solution through employing existing networks of CCTV cameras supported by simple installed beam-crossing sensors.

The development of the method, as well as the collection, analysis, and synthesis of data, was carried out as part of a multidisciplinary team combining specialists in architecture, computer science, electronics and telecommunications, geoengineering, and social sciences. This combination of disciplines reflects the need to provide a multifaceted system for monitoring and analyzing data on users of a unique space such as a university campus. The ultimate goal was the subsequent use of the method in the process of the collection of data on users of other urbanized areas using an existing sensor network, and a proposal for standardizing the ways of processing and presenting of the outcomes. This includes maps visualizing the user flows in the studied area prepared on the basis of acquired imagery data, which can become effective tools for architects and urban planners in the design process.

## 3. Related Works—Research Context

The research context presented below reflects the interdisciplinary team’s work and its multifaceted context corresponding to the needs of the design process, as well as the various competencies of the team members.

### 3.1. Smart City Definition

Millard in [12] defines a smart city as a city in which public issues are solved using information and communications technology (ICT) with the involvement of various types of stakeholders working in partnership with the city’s authorities. In the paper titled “Smart Cities Study: International Study on the Situation of ICT, Innovation and Knowledge in Cities” [13], a smart city is defined as one that makes use of information and communication technologies to increase the interactivity and efficiency of urban infrastructure and its components, as well as to raise awareness among the residents. A similar approach to the issue is manifested by N. Komninos, who defines a smart city as a territory with a high capacity for learning and innovation, that is creative, has research and development institutions, higher education institutions, digital infrastructure, and communication technologies, as well as a high level of management efficiency [14]. According to this approach, it is important to distinguish between a modern city and a smart city. A similar approach is that of A. Caragliu, in whose opinion a smart city is one in which investments in human and social capital and in traditional (transportation) and modern (based on telecommunications and information technology) infrastructure drive sustainable economic growth and build a high quality of life, with wise management of natural resources through so-called participatory management [15].

### 3.2. Revitalization/Planning of University Campuses

University campuses, which are urban complexes built by higher education institutions (HEIs), play an important role in shaping cities, both historically, as important elements of the existing structure, and in the context of its formation and planning. They are also a catalyst for social and economic relations on a local and global scale. Systematic research on the structure of campuses was undertaken only quite recently, at the end of the 20th and beginning of the 21st centuries [16], and identified their key development trends [17]. These trends use formal classification based on morphology, such as the one undertaken by B. Edwards, which indicated nine possible types of campuses: place-making: building-dominated; place-making: landscape-dominated; collegiate; linear; grid; modular; molecular; radial; and ad hoc [18]. Other classifications are based on formal typologies—for example, according to the criterion of the stage of the development process: campuses under construction—contemporary setup; existing campuses being completed (often historical); campuses with a closed spatial structure; and campuses at the conceptual stage [19]. HEIs’ campuses are also the object of research into their planning phases, where one can distinguish educational programming, spaces programming, master planning, and detailed design. This approach stems from the intention to link new functions of university education and research with their corresponding spatial solutions [20].

The factors that shape contemporary academic spaces are closely linked to the new functions of universities that stem from their so-called third mission (known as the triple helix concept) that identifies the university as an equal, third partner in the hitherto bipolar industry–government relationship [21]. The resulting challenges relating to innovation, social problem solving, and the idea of an “entrepreneurial university” imply the need for openness and cooperation with a broadly defined environment. This affects the spatial structures and the development of campuses, including the ways to revitalize the existing ones [17]. An approach that stems from the perception of a university as an efficient knowledge-producing enterprise often leads to the corporatization of academic spaces. This results in the use of solutions and typologies derived from business models, such as strict zoning and access control, as well as designing lounge spaces [22].

The factors shaping academic spaces also include the profile of the student of the future (learning from peers in a peer-to-peer model), changing the forms of teaching (problem-based learning model, balance between on-site and off-site learning, and teamwork), globalization, and the introduction of new user groups resulting from, among other things, the lifelong learning strategy [23,24]. The latter factor strongly implies the need to redefine the profile of the mass campus user and to take into account more people who may have specific needs. This requires addressing these needs within the framework of inclusive design guidelines.

Previous studies have also pointed out the impact of the shaping of campus space on the strengthening of socio-economic ties with the neighborhood and the city [25], which also requires a universal design approach and the use of solutions that are friendly to everyone. The impact of the COVID-19 pandemic is also not negligible, as the design or revitalization of a campus requires maximum flexibility of the architectural measures used (e.g., the possibility to hold classes outdoors). The atomization of relations caused by the pandemic also indicates the need for spatial solutions that facilitate the introduction of integrating social functions.

### 3.3. IoT and Use of Sensor Networks in University Campuses

Sensors that use the IoT technology and networks composed of them—Wireless Sensor Networks (WSNs)—are widely used to collect and analyze data in areas such as agriculture monitoring [26], environment monitoring [27], health monitoring of the elderly [28], industry, especially according to the Industry 4.0 concept [29], smart home systems [30], smart cities [31], and many others. IoT devices are usually small electronic devices that have interfaces for access to the Internet (Wi-Fi, Ethernet, LoRa) or for direct communication (Bluetooth Low Energy, NFC) and allow flexible use for measuring various parameters. The literature identifies works on the use of CCTV in urban spaces [32], public places [33], metropolises [34], suburbs [35], urban parks [36], or public transit systems [37]. Only a few papers deal with the use of CCTV on university campuses. Design research conducted at the University of Technology Sydney showed how CCTV cameras can help interact and manage public spaces [38]. The author presents the potential of CCTV cameras as a design tool. The article discusses how the use of the interactive potential of the CCTV system can influence a new understanding of urban space, which will replace the symbolic role for form with the effect of form.

The other two papers are concerned with security issues on university campuses through the use of CCTV cameras. The study [39] shows that CCTV plays a crucial role in facilitating social interactions and ensuring safety when walking around the dorm at night. The second describes CCTV as a tool providing surveillance at the university and as a critical technology for designing a smart school/campus, as described in [40].

In the literature, we can also find papers that present the application of various methods to design guidelines for university campuses. In paper [41], the application of the Quality Function Deployment (QFD) method was presented. Paper [42] contains case studies on the use of Digital Twin technology in various universities. However, there is no paper in the literature that presents the theoretical framework and practical application of IoT sensors and spatial data mining for analysis of urban space actors’ behavior in university campus space design. We can only find papers that refer to selected aspects of IoT that can be used in designing university campuses, e.g., individuals with disabilities [43].

For the research, beam break sensors with pulse modulation at a set frequency of 1.92 kHz were used. This type of sensor enabled the system to operate effectively under various conditions, regardless of whether it was day or night. Additionally, the sensors operate within the 850 nm wavelength range, which is invisible to the human eye, ensuring both invisibility and energy efficiency. The use of this wavelength also prevents interference with natural light, further enhancing the system’s performance [44].

Data gathered with IoT devices needs to be analyzed and meaningfully presented in order to be useful for design purposes. Spatial data mining, spatial databases, and big data, sometimes referred to as spatial big data, are interdependent technologies, which are used for this purpose [45,46,47]. The main aim of the data mining process is to extract useful information from the data and mold it into an understandable structure for future use [48]. Spatial data mining is the discovery of interesting relationships, characteristics, and patterns that may exist implicitly in spatial databases and data sets [49,50,51].

Based on the selected papers (Table 1), it can be concluded that the interest in the topic of IoT application on university campuses regarding aspects of universal design started in 2016 and continues today. It is noteworthy that the research is mainly conducted in Asia (5), Europe (4), and North America (2). The country with the highest number of studies in this area is Spain (3). The scope of conducted research (Table 1) is quite wide and can be divided into three main categories:

- Models/frameworks of a sustainable and smart campus using IoT technologies [52,53,54];
- Solutions/applications using exponential technologies, enabling more sustainable university campus operations [55,56,57];
- Design and development of infrastructure/technical systems for better campus management, also in the context of universal design issues [58,59,60].

### 3.4. Research Context Summary

A review of the literature indicates that each of the topics analyzed has been the subject of independent research and publication. The novelty proposed in this article is the synergy of the proposed solutions and the use of relatively low-cost, existing infrastructure. The authors’ conceptual and technological solutions allow the use of CCTV camera networks and simple beam intersection sensors for complex spatial analysis to support the process of universal design and revitalization of a university campus.

Although there has been considerable research on using IoT technologies in university campus design, the current literature has notable gaps. Most studies focus on specific aspects, such as security monitoring [32], energy management [58], or systems for supporting individuals with disabilities [56].

Existing research often lacks a holistic perspective that combines technological tools with an understanding of social interactions and how campus spaces are used in practice. Moreover, there is little discussion on how to translate the results of these analyses into practical design strategies, which limits their real-world impact. Another issue is the absence of clear standards for spatial data analysis and the difficulties in integrating various data sources, such as GIS systems, IoT devices, and statistical models.

Addressing these limitations requires further research to develop cost-effective IoT-based approaches that can be seamlessly applied in the design of campus environments.

## 4. Case Study—WUT Campus

### 4.1. Smart City

**Figure 1:**
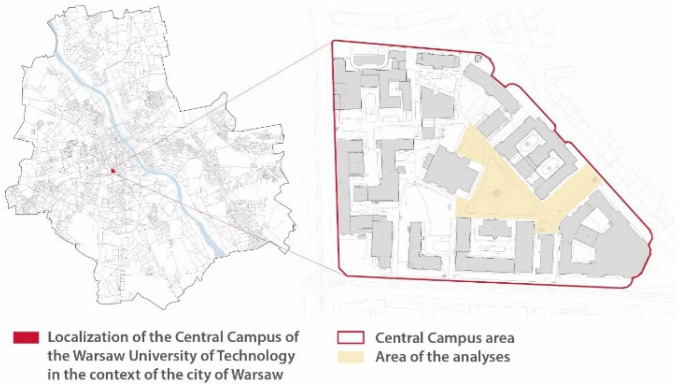
An urban map illustrating the campus location within the city, accompanied by a campus map highlighting the specific area where measurements and analyses were conducted.

**Figure 2:**
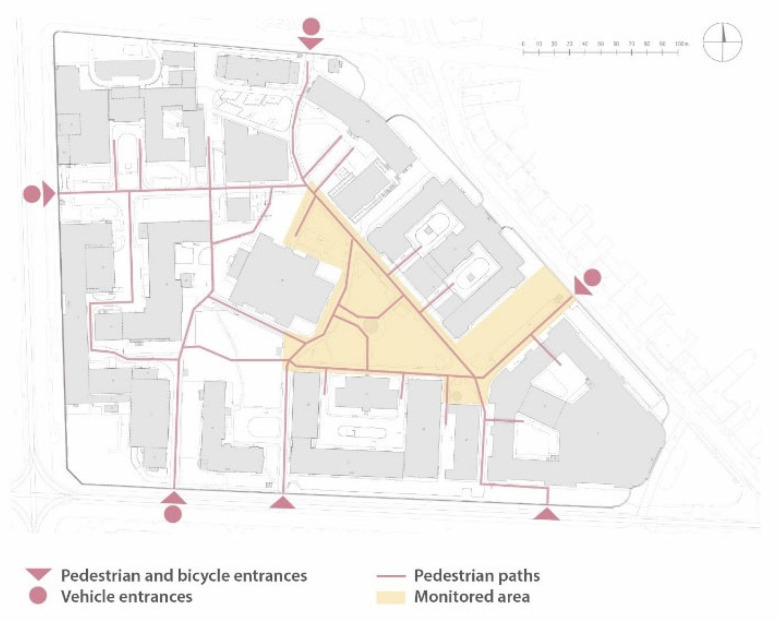
A simplified map of the Central Campus of the WUT, with pedestrian and bicycle entrances and vehicle entrances marked.

The Central Campus of the Warsaw University of Technology is a fenced area located in the central part of Warsaw with an area of 8.88 hectares (Figure 1). The area is a semi-public space which is open to all users but is specifically dedicated to the academic community and the inhabitants of the residential buildings located in the area (Figure 2). There are a total of 17 buildings in the area in question, 15 of which house a total of 10 faculties of the Warsaw University of Technology. The university’s buildings include those built in the initial period of existence of the campus from 1899 to 1939, which were partially rebuilt in the post-war period, as well as later additions built in a style representative of the period of their construction. The buildings most recently were completed between 2010 and 2013. According to the typology presented earlier in this paper, the analyzed area is a building-dominated area (place-making—building-dominated) [18] and can be defined as an existing campus supplemented according to Żabicki’s typology [19]. The site features tall vegetation, including old trees.

### 4.2. Social Studies of the Campus Space

The revitalization of the Warsaw University of Technology campus and the research related to it require the acceptance of the academic community. The campus monitoring and conducting of social surveys were approved by the Warsaw University of Technology’s Senate Ethics Committee, assessing the research as compliant with Polish law. It should be noted that the monitoring images are stored and processed on the university’s servers and cannot be accessed by unauthorized persons.

Research using a variety of surveys (including geo-questionnaires), interviews, consultations, and workshops was conducted by the authors’ team in several phases between 2021 and 2022. An important part of the research was collecting opinions on the receptivity of the university’s student and staff communities, as well as the residents of nearby quarters regarding the use of IoT sensors as a tool for studying social habits, preferences, and needs. This research indicates that the use of some solutions (such as Edge Computing technology) and the application of survey results to universal design increase public acceptance of campus monitoring data collection. The results of the research conducted were published and discussed in [11].

## 5. Methodology

### 5.1. Proprietary Methodology

The harmonization of several processes—the acquisition of image data from CCTV cameras and beam-crossing sensors, the use of deep learning to classify objects in the images, the conversion of the data to a geodetic coordinate system, and the analysis of the data using spatial data mining methods—plays a key role in the proposed research methodology. The analytical process organized in this way enables the conversion of ‘raw’ data from IoT video sensors into structured spatial information. The analysis of this information using geo-information technologies, GIS tools, spatial statistics, and spatial data mining makes it possible to develop spatial knowledge to explain how the space is used by pedestrians and vehicles at different times of the day and week. Equally important is the detection of barriers that impede access to certain places, such as high curbs, sidewalks inaccessible due to improper vehicle parking, blocked escape routes, etc.

The purpose of the conducted research was to assess the feasibility of using IoT sensor data to analyze the functioning of the academic community on the WUT campus. The analysis process used multi-source data and proprietary methods to sequentially process the data into useful information and spatial knowledge:

- use of short-range photogrammetry methods to transform the data from a 2D system (camera frame) to a 3D system (geographic coordinates in campus space). Three different methods were used to transform the data, and the accuracy of the results was analyzed.
- development of a machine learning system. YOLOv8 was used in the developed application, using an extensive learning and validation dataset prepared by the paper’s authors.
- integration of results from beam intersection sensors with results obtained from machine learning methods. The methods developed by the paper’s authors for integrating heterogeneous data enable multifaceted analysis of pedestrian, bicycle, and vehicle activity on the WUT campus.
- spatial–temporal analyses using GIS tools to assess pedestrian and vehicle traffic on campus at different times of the day and week.
- comparison of quantitative results of spatial–temporal analyses with qualitative analysis of spatial distribution of the use of different parts of the campus during the day. The developed methodology was further verified by conducting observations on campus. The spatial knowledge base developed, and the conclusions obtained made it possible to propose changes in the way the WUT campus is used.

**Figure 3:**
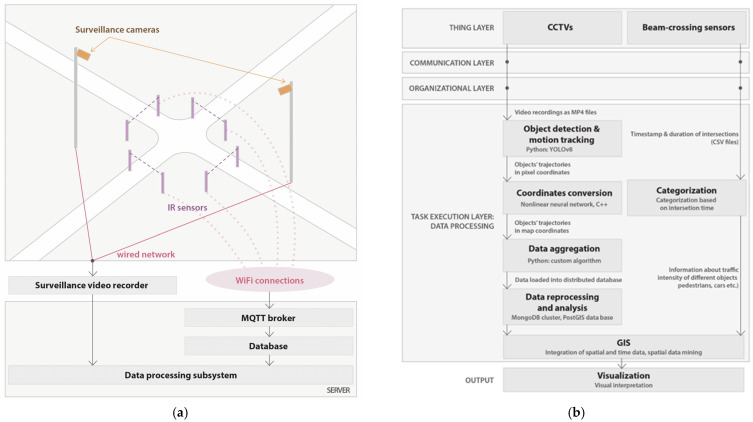
Acquisition process of spatial–temporal knowledge seen from two perspectives: (**a**) our IoT sensor network’s architecture and (**b**) a data processing scheme.

The diagram of the proposed data acquisition and processing methodology is shown in Figure 3. The process of the acquisition of spatial–temporal knowledge based on an analysis of data coming from a network of IoT sensors includes the following:

- Collecting image data from CCTV cameras with image processing and extraction of the derived information about the type of object (e.g., a man, a car, etc.) and its movement parameters;
- Collecting and analyzing data on traffic intensity in key areas of the studied space based on the interpretation of the measurements from beam-crossing sensors;
- Integrating data and derived information from various devices in a NoSQL-type database;
- In-depth data analysis using spatial data mining methods and visualization of the results.

The issue of implementation of the proposed methodology and analysis of its usefulness are further discussed in the following sections of this paper.

### 5.2. Description of the Equipment Used

**Beam intersection sensors.** Detection of urban space actors can be implemented in a number of ways, e.g., PIR sensors (Passive Infra Red, commonly known as motion detector), microwave doppler radars, or Time-of-Flight laser range sensors. Each of these methods can be characterized by its range, reliability, price, energy consumption, and resistance to weather conditions such as intensive sunlight, rain, and snow. The relative weights of these factors also depend on the survey scenario chosen, i.e., whether the analysis is carried out continuously or in short sessions, whether there is access to mains power, and the number of points to be surveyed.

In the discussed case, several weekly measurement sessions were planned at multiple points on the WUT campus at different times of the year. An infrared barrier connected to an ESP32 microcontroller was initially chosen as the optimal solution to build a distributed data collection subsystem for the analysis, mainly due to its low cost, weather resistance and battery operation, allowing a flexible choice of installation locations. Custom-made devices based on ESP32 CAM boards (Espressif Inc., Shanghai, China) and Photocell Sensor Infrared Detector (OEM Tech Sp. z o.o. Warsaw, Poland) IR sensors have been developed. The detection range of the IR subsystem is specified as 10 m at 12 V power supply, which fits our requirements. Microcontrollers communicate over the WUT’s Wi-Fi network available on campus, using the NTP protocol for time synchronization and MQTT protocol to exchange data with a central server for data storage.

**CCTV cameras.** When considering the practicalities of data collection, issues such as installation effort and site selection, limited battery life, risk of theft, etc., need to be taken into account. With this in mind, we examined another source of pedestrian and vehicle traffic data on the WUT campus—surveillance cameras. These devices are attractive for several reasons. Modern neural networks based on deep learning enable reliable recognition and tracking based on recorded video sequences, which opens up possibilities for automated but precise analysis. Surveillance cameras are commonly used in public places, so it is highly likely that additional equipment will not be needed as long as access to their recordings is possible. When analyzing the resulting imaging data, it is, of course, important to remember that the limited view of CCTV cameras may result in missing data in certain areas. The cameras installed at the WUT campus offered high-quality images with HD or HD+ resolution and a frame rate of around 15 frames per second. For the analysis, we chose only fixed-position and fixed-zoom cameras—this is important because object detection algorithms return the positions of objects in the screen coordinate system (the positions are given in pixels in the image frame), and we need to map these points onto a flat topographic map. Such cameras make it easy to define non-linear neural networks for coordinate conversion.

### 5.3. Sources of Input Data

**Figure 4:**
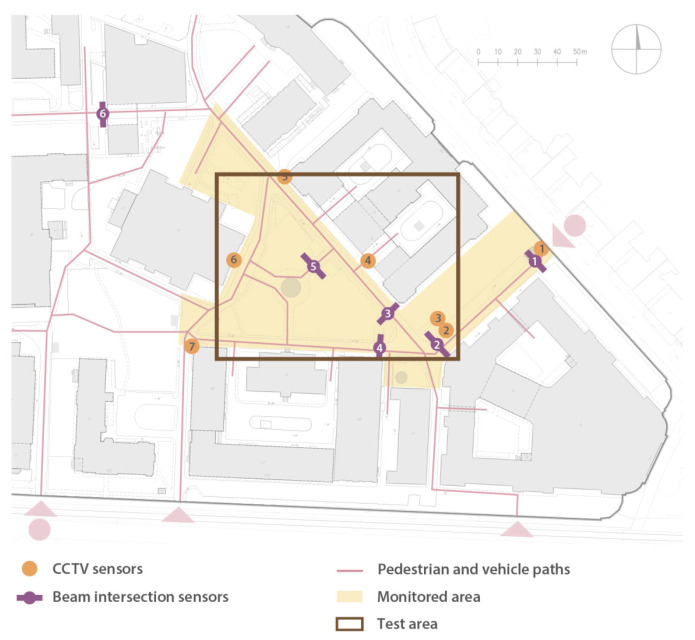
Location of cameras and sensors on a map of the WUT Main Campus.

**Figure 5:**
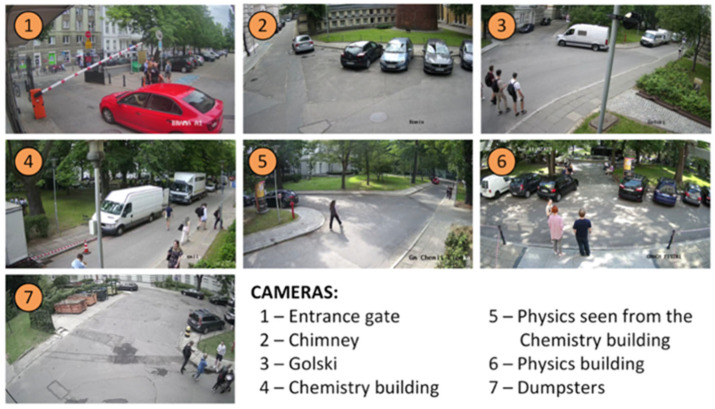
The view from each of the analyzed surveillance cameras.

The measurements carried out between 16 May and 28 November 2022 used six beam-crossing sensors placed on campus, as shown in Figure 4. A rectangular test area of approximately 1.1 hectares (122 by 92 m), colored brown, was selected for the study. The input data are in the form of an array of beam intersection events with timestamps, along with their duration, measured with millisecond resolution. Video data from surveillance CCTV cameras (see Figure 4 for locations, see Figure 5 for exemplary views) were obtained from university campus administration for the following periods: from 7 July to 15 July, 23 October to 30 October, 21 November to 28 November 2022, later considered “sessions”. The analysis sessions were limited to selected weeks due to the large effort required to process the data. To give a point of reference, the total size of all video files acquired from CCTV cameras from the last session (as an example) was about 900 GB, which corresponds to a total of about 1170 h of video footage (7 cameras, 7 full days). The average bitrate per video stream was 1.7 Mbps, with significant variation depending on the amount of recorded traffic. The recordings have been processed according to the procedure described in the next section. It should be noted that operating on video files (rather than real-time data processing) was chosen for this study for practical reasons: this allowed the processing algorithms to be repeatedly improved and analyzed in a controlled environment without losing the overall concept.

### 5.4. Description of the Video Data Processing Method

The data collected during the measurement campaign were processed and aggregated using both standard IT tools (relational type NoSQL databases), GIS packages, and statistical tools, as well as proprietary scripts developed mainly in the Python 3.10 and C++ languages. The main difficulty in the overall workflow is rooted in the wide range of tools and the large number of steps required to make the video a useful data source. The following steps were considered essential: object detection, object tracking, and coordinate conversion. Next, data aggregation and careful filtering are necessary before statistical analysis. Each of the processing steps mentioned is a potential source of errors or inaccuracies.

The first two steps were performed on each video file in an automated manner using the YOLOv8 Ultralytics library [63] with the BYTE object tracker [64]. Initially, the performances of the YOLOv8 ‘Nano’ and ‘Small’ models were compared in a test run. Typical model preparation steps were performed. First, 4652 video frames were selected from all cameras involved in the study, including samples from different times of the year and day. The data also included frames recorded after dark, when the cameras operate in infrared mode. The objects of interest in each frame were then annotated using a web-based annotation platform (cvat.ai). Only one class (‘person’) was included in the trial, and other classes (e.g., ‘bicycle’, ‘car’, ‘truck’, ‘bus’) were added later. The dataset was divided into three parts for training, validation, and testing respectively. The transfer learning approach was chosen on a model already pre-trained on the COCO dataset. The results of this trial comparison (see Table 2) allowed us to conclude that the ‘Nano’ model has about four times fewer parameters, resulting in significantly less computational effort for training and using the model, while the performance metrics are only slightly better for the ‘Small’ network, with differences ranging from 0.1 to 1.7 percentage points. Despite choosing the ‘Nano’ model, the inference process was still extremely time-consuming for such a huge dataset. It was greatly accelerated by using the NVIDIA A100 GPU, which was made available to us by the WUT CENAGIS data center (https://cenagis.edu.pl/ (accessed on 1 December 2024)).

**Figure 6:**
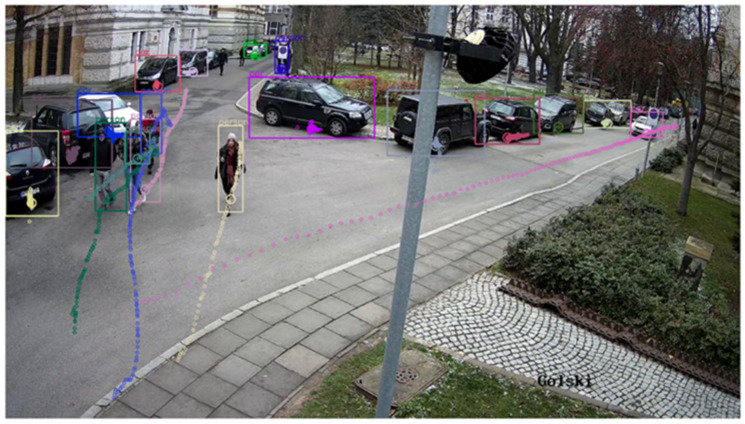
An example of a single frame from the Golski camera with detected objects (pedestrians and cars) being indicated by rectangles. Small circles indicate the trajectories of their movements.

The object detection stage finished with a description of each video frame containing a list of recognized objects, their on-screen coordinates, as well as a set of auxiliary data such as confidence level, frame number, timestamp, etc. An example of a single frame with plotted rectangles around the detected objects and their extracted trajectories is shown in Figure 6.

Using fixed-position and fixed-magnification cameras as data sources, it was possible to convert objects’ coordinates to the PUW 2000 national geodetic coordinate system (zone 7). The coordinate transformation is based on the calibration of the monitoring cameras to a chessboard pattern and the coordinates (in the global coordinate system) of the characteristic points visible on the individual cameras. Calibration and measurement of these points were performed during a separate measurement campaign. Having obtained the calibration matrices and alignment points, a PnP (Perspective-n-Point) transformation was used to obtain the constant (for a given camera) coefficients necessary to convert the camera coordinates to the global system. The developed system has made it possible to obtain a sub-meter transformation error for most objects observable by CCTV cameras. A single point located at half the width and 20% of the height of the rectangle surrounding the object (marked with a small circle in Figure 6) was taken for projection.

**Figure 7:**
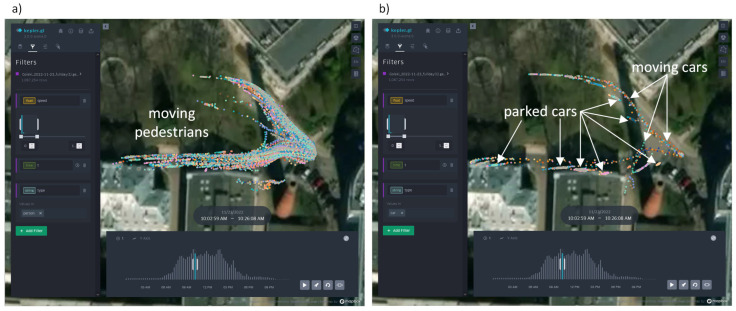
An example of trajectory visualization in the Kepler.gl application: pedestrians (**a**) and cars (**b**).

At this stage, the Kepler.gl online application proved to be a very handy tool for debugging purposes. It accepts trajectory collections saved in GeoJSON files. An example of a small trajectory subset recorded by the Golski camera and visualized with Kepler.gl is presented in Figure 7—separately, pedestrians (a) and cars (b). In this example, some small projection errors are noticeable in the areas located far from the camera due to insufficient calibration—this was fixed later.

### 5.5. Modification of Data Processing

Processing of trajectories obtained from CCTV cameras poses some difficulties due to large amounts of data, which significantly increase requirements for RAM memory and slow down analysis and data visualization. The reason is the frame rate of approximately 15 frames per second, which yields unnecessarily dense motion path mapping. Redundancy is highest for still and slowly moving objects, like parked cars or standing and talking people, etc., so spatial subsampling and temporal subsampling were applied to facilitate further processing, described in the following paragraphs.

**Figure 8:**
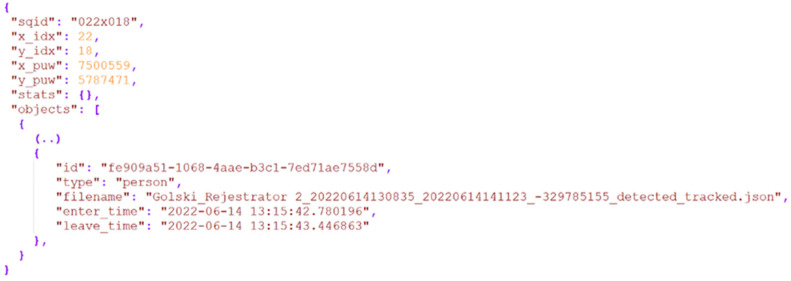
An example of data structure (presented in a JSON-like format) describing the passage of a person through a square with X, Y coordinates: 7500559, 5787471.

The spatial subsampling was implemented by dividing the area in question into a regular matrix of squares measuring 2 × 2 m. Each square was assigned the camera that offered the fewest detection errors. After the coordinate system conversion, each point from the trajectory of each detected object was classified as a visitor to that square, depending on its geographic coordinates. Data were collected on the visits of each object in each square, including the identifier, entry time, exit time, and object type (see Figure 8 for an example). After loading the data into the Mongo DB database, several statistical measurements were calculated for each quadrant, which were then visualized on a map. These measurements included the number of objects visiting each square, the total duration of all visits to the square, separate calculations for different types of objects (pedestrians, bicycles, cars, and trucks), detection of non-moving objects (such as parked cars), and the daily variability of these parameters.

Interesting analyses can be performed on a trajectory basis instead of the spatially subsampled “visits in squares” described above. For this purpose, trajectories were processed by the MovingPandas Python library [65], which offers several valuable processing algorithms, like calculation of velocity, direction of movement, intersections, as well as enabling easy data exchange with other GIS tools using built-in exporters to, e.g., GeoJSON data format.

The spatio-temporal data processing methodology developed by the authors was inspired by the work of [66,67]. When processing trajectories using the MovingPandas library, practical problems related to long processing times or high RAM requirements can easily arise. The spatial subsampling technique mentioned earlier cannot be applied, because many interesting details could be easily lost, or the trajectories could be disturbed. Instead, temporal subsampling was used, removing 7 of every 8 data points from all trajectories. This reduced the dataset by a factor of 8 without noticeably degrading the representation of the trajectories. A limitation of temporal subsampling is the maximum velocity of moving objects (e.g., fast-moving cars)—it is important to make sure that such an object is not completely removed after subsampling, and its speed information can still be estimated. Also, trajectories shorter than one second have been completely removed from the analysis, as they are usually the result of poor object detection, e.g., caused by occlusion or a large distance from the camera. To further speed up processing, it makes sense to minimize redundancy and remove unnecessary data. One useful solution turned out to be the MovingPandas’ built-in TrajectoryStopDetector class, which helps detect and remove non-moving objects (such as parked cars).

### 5.6. Data Synchronization Between Beam-Crossing Sensors and CCTV Cameras

Another type of analysis is the cross-validation of results between those based on CCTV camera data and those based on beam-crossing sensor data. The consistency of the results obtained by two completely different methods potentially provides a high level of confidence for the overall methodology. While such a comparison may seem trivial at first glance, in reality it is not. The reason is the need for good time synchronization between the two data sources. Correct timestamps assigned to individual events are essential. The beam-crossing sensors were synchronized via a Network Time Protocol (NTP) with a time server accessible via the Internet at the time of installation. In practice, a relatively small (a few seconds per day) error was observed due to temperature drift of the quartz oscillator on the ESP32 board, but this is relatively easy to fix with repeated synchronization with CCTV-derived data.

The situation is a bit more complicated for CCTV cameras. First of all, since the video recording system used by the CCTV cameras is controlled by the university administration, it was difficult to obtain reliable confirmation of whether the NTP server is used as a time-synchronization method. To make the methodology more general, it was assumed that NTP might not be used, so an unknown time offset might be present in the timestamps of all video files. Secondly, a video file usually contains a timestamp only in the file name, indicating the start and end time of the recording, which entails calculating timestamps for each frame of the video separately. Third, in the video files obtained, it was found that the duration of the video frames was not constant, making it impossible to accurately calculate timestamps based on the averaged FPS value.

It was considered impractical to recognize timestamps using OCR techniques based on embedded textual tags in the video content. Instead, the timestamp of each video frame was extracted from the input video data stream using low-level techniques. The Decord Python library (Decord v.0.6.0) was successfully used for this purpose. The key point was to read for each video frame its property characterizing its duration and based on this, calculate the current timestamp. In this way, the accuracy of determining the time of events in the video relative to its beginning was obtained in the order of tens of milliseconds, making this problem negligible in practice.

**Figure 9:**
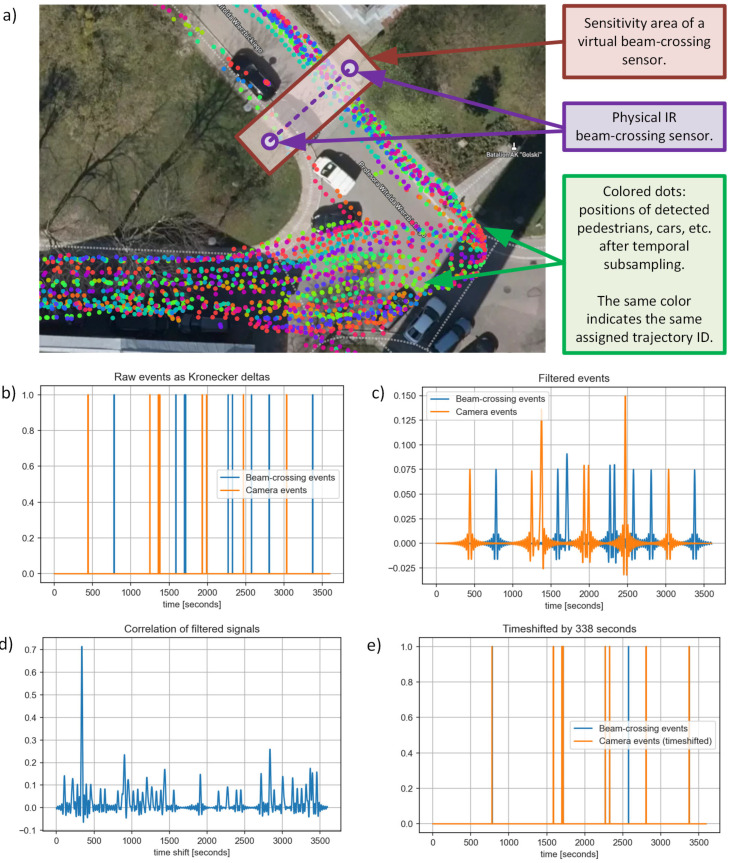
Explanation of the technique to automatically remove the time shift between beam-crossing and CCTV sensors: positions of physical and virtual beam-crossing sensors (**a**), initial signal vector created from detected events (**b**), both signals after low-pass filtering (**c**), result of cross-correlation between those signals (**d**), events from both sensors after time alignment (**e**) (“Golski” camera, 26 November 2022, 6:00–7:00).

However, the unknown time offset between the beam-crossing sensors and CCTV cameras may still be present in the measurement data due to the lack of global time synchronization during the measurement session. As a solution, an automated approach for estimating the time shift was implemented. The method is based on cross-correlation calculated for two signals in the time domain. One signal represents the events of passing objects (people, cars, etc.) through a physical IR beam-crossing sensor. The other represents the same events, but the information is extracted from the video analysis subsystem as the intersection of the trajectory of detected objects, with a polygon representing the approximate sensitivity area of a “virtual beam-crossing sensor” located in approximately the same place as its physical counterpart (see Figure 9a). The timestamp of each event is calculated as the average value between the time of entry and exit from the area. The two signals are constructed in a similar manner. First, the sample rate is arbitrarily chosen as 1 sample per second. Both signals are created as zero-filled vectors with a length equal to the selected correlation length, such as 1 h (which corresponds to 3600 samples). Then samples at time positions corresponding to the time of the above-detected events are set to one (in other words: Kronecker deltas are inserted at these positions). An example is shown in Figure 9b—a fixed time offset between the two signals is visible and must be calculated. While the positioning of the virtual beam-crossing sensor on the map is not ideal, nor are the results of the entire video processing, including tracking and projecting objects’ positions on the map, which created some errors, through which direct calculation of the cross-correlation with such signals resulted in poor accuracy and confidence in estimating the time shift. A significant improvement was achieved after applying low-pass filtering, which expands the pulses (see Figure 9c). Finally, the timing of a single highest peak in the cross-correlation results is selected as the estimated time shift (Figure 9d). Once the time offset is eliminated, the two data sources are synchronized, and detection events can be analyzed on a common timeline (Figure 9e).

## 6. Examples of Results

### 6.1. Intermediate Results

The measurements were performed using beam-crossing sensors in six locations (see Figure 4) in the period from 16 May to 28 November 2022, in five campaigns. More than 140,000 individual measurements were collected, covering 1066 h. The objectives of the first campaign were to identify the beam intersection timing for pedestrians and to estimate the value of beam intersection by a single person, since it was intersected by a group of pedestrians or a vehicle. On 16–17 May 2022, 7609 measurements were performed on the narrow sidewalk at one of the campus entrances (17 h) at Noakowski Str.

**Figure 10:**
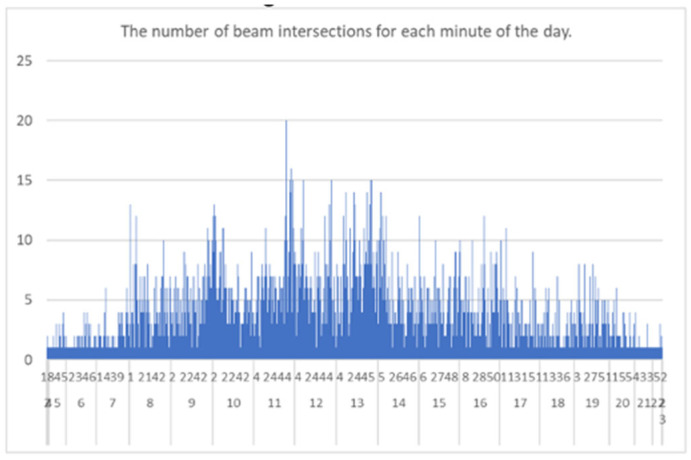
The measurements performed by the beam-crossing sensor installed in location 2 (see Figure 4) on 13 July 2022 (Monday, during a regular semester).

Figure 10 shows the measurements performed by the beam-crossing sensor installed in location 2 (see Figure 4) on 13 July 2022 (Monday, during a regular semester). The results show heavy traffic at 8:00 AM, 10:00 AM, and 12:00 PM (most classes start at 8:15 AM, 10:15 AM, 12:15 AM, 2:15 PM, 4:15 PM, and 6:15 PM). Moreover, traffic mainly occurs between 8:00 AM and 8:00 PM—during class hours.

**Figure 11:**
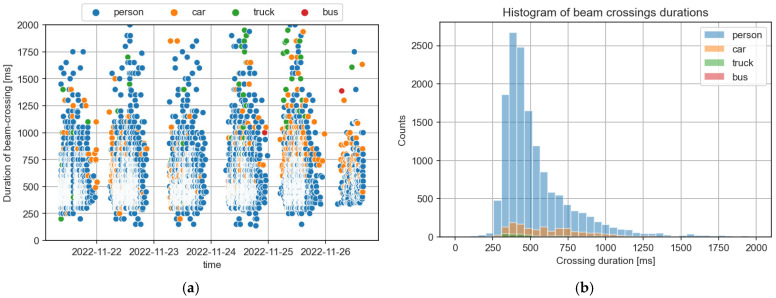
Individual durations of beam-crossing events as a function of time and object type (**a**), and aggregated into histograms for pedestrians, cars, trucks, and buses (**b**). Based on data from beam-crossing sensor (location “0”) with assigned object type from “Golski” camera sensor, acquired 21–27 November 2022. Events without assigned object type as well as those with durations longer than 2000 ms have been truncated from analysis.

Although beam-crossing sensors are convenient due to their low cost and easy installation, they provide a limited ability to distinguish between different object types. Using synchronized event data from CCTV, detected passes were recognized and labelled using AI technology, as described above. For each event detected by the beam-crossing sensor, the same object type was assigned as the event detected in the CCTV pipeline with the same timestamp (a small tolerance of 2 s was allowed for system calibration uncertainty). The results are shown as dots in Figure 11a, where the x-coordinate corresponds to the timestamp of the event and the y-coordinate is the measured duration of a given beam-crossing event. Combined with Figure 11b, which shows a histogram of beam-crossing durations, it can be concluded that it is virtually impossible to use the duration of the beam-crossing event as a differentiating factor between object categories, as was initially considered.

The example presented above demonstrates the power of cross-validation between different sources of data acquisition and processing techniques. First of all, a relatively good timing synchronization has been achieved. Agreement in results obtained with two completely different methods potentially gives a high confidence level for the overall methodology. This opens the doors to many interesting analyses which can be performed. Obtained datasets can be analyzed together and cross-validated against missing data, false-positives, etc. Beam-crossing sensors provide just the timing and duration of the crossing event. The camera-sourced data were used to verify whether any additional information can be derived from the measured durations.

### 6.2. Pedestrian Traffic

**Figure 12:**
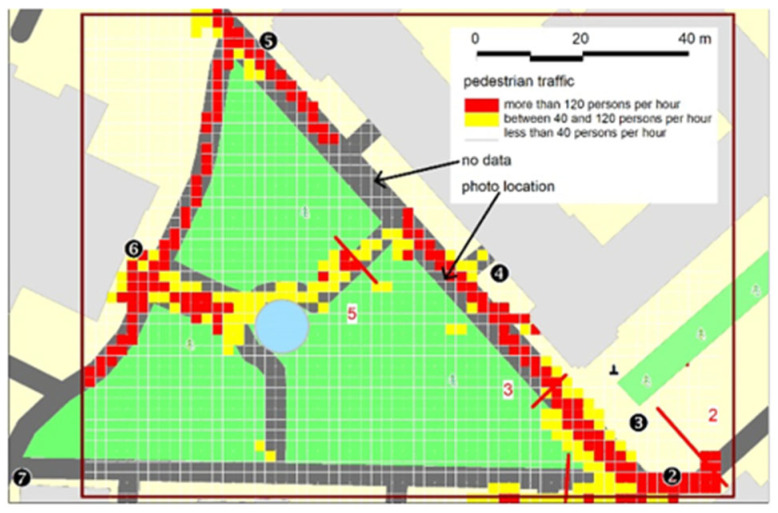
Pedestrian traffic intensity on 14 May 2022.

**Figure 13:**
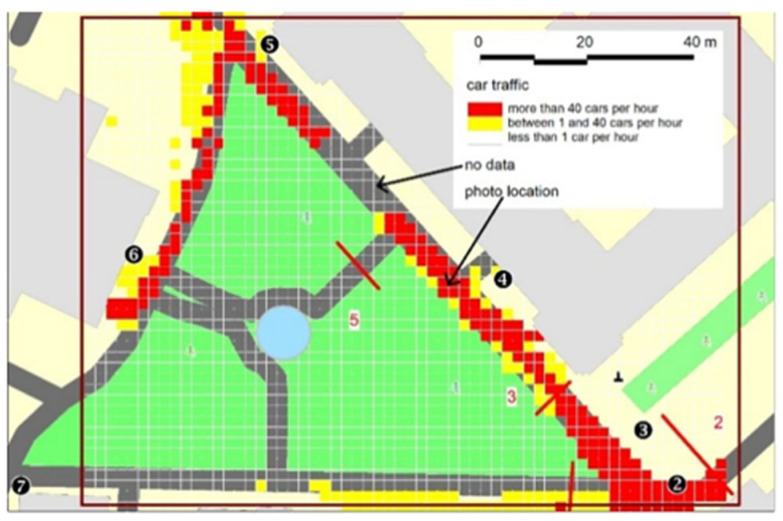
Car traffic intensity on 14 May 2022.

The illustrations in Figure 12 and Figure 13 indicate that more than 120 people per hour move along this tract and more than 40 cars pass by. It should be noted that due to the improper parking of vehicles on the sidewalk, pedestrian traffic largely travels on the street intended for vehicular traffic. This is confirmed by both the analysis of the result maps (negligible pedestrian activity on the sidewalk along the lawn) and the photo from the “Gmach Chemii” (Chemistry Building) camera, indicating the dysfunction of this part of the campus (see Figure 4, location 4).

In addition, the data obtained from the analysis of camera images made it possible to confirm the proposition on the dysfunctional nature of the space use that results from the excessive number of vehicles entering the central part of the campus. The measurement methods showed that the sidewalks in the central area of the WUT are blocked by the cars parked there (this was shown by the measurements carried out by the beam-crossing sensors at locations 3 and 4) and that the users in that area move on the roadway (e.g., images from camera no. 3). This provides a tangible argument in the discussion on limiting the zones allowed for car traffic at the campus and limiting the total number of parking spaces.

**Figure 14:**
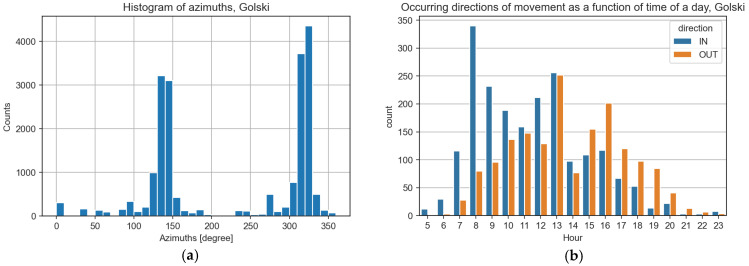
Directions of movements on the beam-crossing sensor location 3, estimated by camera “Golski”.

Trajectories measured with cameras can be analyzed against the direction (azimuth) of movement and velocity of objects. In Figure 14a, a histogram of azimuths detected at sensor location “3” and observed by the Golski camera is presented. It is clear that movement can be easily categorized into two categories: “IN” (as objects entering the interior of the campus) and “OUT” (as opposite). Categorized events from a single day counted in one-hour periods are presented in Figure 14b, which confirms a natural situation to enter the campus mostly in the morning and leave it in the afternoon hours.

As a result of the analyses conducted, a comprehensive set of numerical data was collected, representing both pedestrian and motorized users within the analyzed section of the Central Campus of Warsaw University of Technology. This set of data includes detailed information on traffic volume at different times of the day, allowing for an accurate assessment and comparison of the number of pedestrians and vehicles in selected locations on the campus.

### 6.3. Impact on the Campus Revitalization

The information obtained from beam-crossing sensors and the video analysis from surveillance cameras resulted in an input to refine the design. Information on the acquired data and the resulting guidelines for the next design stage are shown in Table 3.

The data collected made it possible to verify the design assumptions in order to optimize the width of pedestrian and pedestrian–vehicle routes, to place seats, to place information elements, and to select locations for potential attractors.

In this study, a spatial analysis model leveraging IoT technology was developed to enable the systematic collection and analysis of data on spatial usage patterns. The model employs a structured approach by dividing the analyzed space into nodal points (key locations for traffic flow, such as intersections) and edges (connections between these nodes). This framework facilitates the examination of user numbers, movement directions, and speeds within the analyzed area.

CCTV footage has proven particularly valuable for the analysis of nodal points, as it not only provides detailed data on traffic flow but also supports the categorization of users into distinct groups, including pedestrians, small vehicles (e.g., bicycles, scooters), and cars. Such categorization enhances the applicability of the results to urban planning and infrastructure design. Basic throughput data for individual edges can be obtained using beam-break sensors, which provide direct input for decisions concerning the design and optimization of pathway widths. However, the study revealed that beam-break time in sensor-based systems are not directly correlated with the type of object crossing the beam, highlighting the importance of complementary data sources.

The application of the model on the WUT campus enabled the identification of the most heavily utilized routes and potential directions for spatial revitalization, contributing to the enhancement of campus functionality. The versatility of the proposed model allows its adaptation to a wide range of urban environments, including city centers and recreational areas. Furthermore, the dynamic analysis of movement patterns opens pathways to advanced applications, such as traffic forecasting and anomaly detection, fostering the development of more efficient and functional spaces.

## 7. Discussion

### 7.1. Data Acquisition

The user movement data acquired from the sensors at the design detailing stage made it possible to verify the design assumptions made and to develop the recommended changes that are feasible at the current stage of the design. At the same time, there is an argument in the discussion on the way to adopt the final version of the design, using participatory tools.

When analyzing the resulting information, it is also important to note the area of missing image data (indicated by the arrow in Figure 12 and Figure 13). While cameras 3 and 4 provide full imaging coverage of the southern part of the analyzed road, the field of view of cameras 4 and 5 prevents full analysis of the northern part of this street. Also, camera 7, located in the southwest corner of the test area, faces north and does not provide full image coverage of the southern part of the area. However, due to the well-defined spatial arrangement of roads and paths, it is possible to interpolate temporally and spatially the missing parts of the image. In further studies, however, it is advisable to densify the sensor network, especially CCTV cameras.

In the future, receiving data at the initial design stage can contribute to a faster and, at the same time, more efficient and in-depth preliminary analysis of the designed area. Obtaining detailed information on the characteristics of the use of the area, broken down by type of users and hourly distribution throughout the day and week, provides an opportunity to thoroughly assess the current degree of use of the area, including identification of its strengths and weaknesses. At the concept development stage, the results obtained can make a significant contribution to an increase in inclusivity, elimination of existing terrain obstacles, and increase in the efficiency of the use of developed urban areas.

The data acquisition technique based on CCTV cameras, although attractive due to its flexibility and relatively large coverage area per device, is prone to a number of practical problems. In the described prototype setup, we experimented with already installed surveillance cameras with offline analysis of the recorded video files, as well as with live analysis performed by a dedicated camera provided with object recognition algorithms. The second approach follows the edge computing concept; however, it requires more effort to install the devices. An added benefit is that it does not require time-consuming postprocessing of the recordings, because data about the position of objects are obtained directly during observation.

Should the process be carried out in any other circumstances and locations, the data privacy question is to be taken into consideration, since regulations and image content may vary. The general rule of CCTV systems is that images are stored locally for at least some time, so if they are not transmitted elsewhere, the question is mainly how to provide sufficient control over images access while processing them and to resolve longer-term storage, if it is applicable.

It needs to be considered that privacy issues may affect the possibility of rendering such datasets accessible for testing research repeatability; however, this would be the case for any CCTV images containing people.

### 7.2. Data Processing and Analysis

An analysis of the collected data demonstrated that the use of a system of surveillance cameras is sufficient to develop complex models of time- and space-varying use of campus space by pedestrians, cyclists, cars, and delivery vehicles. Image data processed using proprietary scripts and analyzed using spatial data mining methods enable analysis of social behavior, detection of spatial dysfunctions, and indication of their location, as well as optimization of the architectural design method. Due to the speed of the change associated with the intensity of traffic, an analysis of the dynamic image may cause classification uncertainties—for example, in the recognition of objects—but this does not have a significant impact on the statistical analysis of the collected data. Similarly, due to the uneven distribution of transformation points in individual images and the associated insufficient training of neural networks in the process of conversion of image data to GIS spatial database structures, there are occasional errors in assigning object motion to the wrong basic field. However, the use of filtration of results using topographic data makes it possible to eliminate information noise and analyze the data aggregated to basic fields with a resolution appropriate for the purpose of the research. Further work on a modification of the developed technology will involve the development of proprietary software for the purpose of full automation of the processes of data processing and knowledge acquisition in the edge computing mode without the need for post-processing.

### 7.3. Broader Implications for Smart Cities and Participatory Urban Design

The methodology described in this study builds on established concepts of smart cities as defined by Komninos et al. [14] and Caragliu et al. [15], which emphasize the integration of information technologies to support participatory urban management. Our approach extends these frameworks by introducing a scalable system for monitoring and analyzing spatial behaviors using low-cost IoT infrastructure. Unlike studies focusing solely on technological implementations, such as LoRaWAN-based energy efficiency [58,59], our methodology bridges technical applications with participatory design processes, making it directly relevant for inclusive urban planning. The methodology can be adapted to resource-constrained environments, providing valuable insights into space utilization and urban behavior patterns.

The prototypical methodology presented in this study demonstrates its scalability and adaptability for various urban settings beyond university campuses. Unlike previous studies that often focus on specific technologies or narrow applications (e.g., energy management [58] or security [40]), our integrated approach bridges the gap between theoretical frameworks and practical implementation in the field of participatory urban design.

The study shows how real-time data related to urban space actors’ behavior can create a stronger connection between urban planning and the people it serves. Cities thrive when technology supports meaningful citizen involvement, as highlighted by Azkuna [13] and Millard [12]. By focusing on practical, scalable methods, the findings illustrate how urban spaces can be renewed in ways that are not only inclusive but also sustainable for the future.

The authors’ findings align with prior research on the integration of IoT and spatial data mining in urban design contexts (e.g., [12,45,46,47]). Similar to previous studies emphasizing the utility of IoT in optimizing resource allocation [52,53,54], our results validate the effectiveness of low-cost sensors in providing actionable data for space design. However, our study extends the scope of earlier works by demonstrating a novel methodology that combines beam-crossing sensors and CCTV data for the revitalization of university campuses. This contributes to the discourse on data-driven urban design by offering a scalable and efficient framework for analyzing pedestrian and vehicular flows in complex urban environments, which has not been explored in detail in prior research [56,57,58,59,60].

Unlike traditional approaches focusing solely on data collection, our method integrates real-time spatial data mining to provide insights into behavioral patterns over time. This approach bridges the gap between empirical observations and their practical application in campus design, aligning with Komninos’ theoretical framework on smart city innovation [14]. Furthermore, the findings contribute to the emerging body of work on the role of technology in enhancing deliberative democratic processes, as suggested by Caragliu et al. [15].

## 8. Conclusions and Future Work

The method proposed and analyzed in this paper, which utilizes data on the use of the existing urban fabric and IoT sensor networks, has shown several benefits for the design process, such as, for example, indicating the dysfunction of certain traffic routes, the capacity of the routes, and the actual state of their use. It also provides the ability to observe measurements and accurately determine the temporal and spatial aspects of architectural fabric use. According to the authors, this greatly benefits the possibility of verifying the assumptions of the campus revitalization design.

The proposed four-layered architecture of the IoT system was detailed and tested on a prototype of the measurement and computing system. The measurements collected were used to make changes and refinements in the architectural design.

The research indicates that the data collected and analyzed can be used during the work on the campus development concept. The design assumptions, based on the knowledge and experience of the designers, as well as on the analysis of analogous case studies, can be supported by experimental data, the analysis of which influences the refinement of some of the recommended solutions. The results of the research also support the implementation of changes and provide a solid basis for the arguments of their advocates.

The implementation of campus monitoring technology that uses IoT sensors concerns members of the academic community and passersby who use the university’s area. The research conducted has demonstrated the effectiveness and efficiency of the proposed methodology, but the application of a remote monitoring technology that uses IoT sensors requires public acceptance. Another important role for the decision-makers who decide on the development of the campus is to be aware of the impact of the protection of the personal data of the campus space users. People are concerned about the potential for misuse of this technology. If it is to be deployed, an awareness-raising campaign is needed to increase the support for new investments as well as technical measures, such as edge computing, which have to be adopted.

Although the methodology remains in a prototypical phase, it demonstrates significant potential for application in broader urban contexts. By building on frameworks for spatial data analysis, such as those described by Ng and Han [50], this research creates a replicable model for urban revitalization projects. The focus on using existing infrastructure, including CCTV networks, aligns with the cost-effective and resource-efficient approaches advocated by Ogrodnik [4] and Zavadskas et al. [5]. Future work should automate the data processing pipeline and explore its application in larger urban areas.

The method presented in the paper is prototypical and requires many improvements. An important issue is the work on the automation of the communication between sensors, online data transfer, securing devices against cyber-terrorist attacks, and connecting all sensors into a single network.

Limited access to resources also makes it necessary to search for solutions that minimize the installation and maintenance of new sensors and that are based on existing infrastructure, such as a network of surveillance cameras. The developed data acquisition and processing method can be used universally to study user behavior in any area covered by a surveillance system, taking into account the specific characteristics of the local hardware.

A university campus is a natural testing ground for introducing the smart city concept and testing new solutions, including in the context of the current trends in the development of HEI spaces. The interdisciplinary research conducted is an attempt to take advantage of this opportunity, as well as to develop a methodology for the performance of revitalization tasks in university areas.

## Figures and Tables

**Table 1:** Characteristics of papers on the impact of IoT sensor networks on the design of university campuses.

Paper	University/Country	Technologies Used in the Study	Contribution
[11]	Warsaw University of Technology/Poland	IoT, edge computing	key factors influencing the acceptance of IoT devices for universal design
[52]	Izmir Bakırcay University/Turkey	cloud, IoT, big data, mobile, and AR	roadmap for establishing a smart and sustainable campus; framework including an architectural structure and the application process
[53]	UKM Bangi/Malaysia	IoT, LabVIEW, wireless connectivity, Universal Software Radio Peripheral	smart campus model provides learning process availability anytime and anywhere to assist students and lecturers
[54]	University campus as a research object	IoT, big data, Hadoop, cloud computing	model of a sustainable smart campus using IoT, big data, centralization and analysis of data—application of smart city model (from macro to micro scale)
[55]	Shandong Normal University/China	IoT, OAuth authentication, data analysis platform, GIS, cloud computing	smart campus application system with existing educational resources
[56]	King Saud University/Saudi Arabia	wireless sensor network, GIS, RFID, SQL	university-based smart and context-aware solution for people with disabilities
[57]	University of Washington/USA	IoT, machine learning, Raspberry Pi	use machine learning techniques to develop a method for the dynamic identification of parking space topologies based on the position of parked vehicles
[58]	University of Murcia/Spain	data mining and analytics, big data, IoT, ICT	Energy-Aware IT Ecosystem Architectural Approach—the ENTROPY IT; supported energy management, positive impact on energy efficiency
[59]	University of A Coruña/Spain	IoT, fog computing, 3D ray-launching, wireless sensor networks, cloud, LoRaWAN	design and deployment of a LoRaWAN IoT-based infrastructure able to provide novel applications (i.e., mobility pattern detection system, smart irrigation solution, smart traffic-monitoring deployment) in a smart campus
[60]	University of A Coruña/Spain	IoT, LP-WAN, LoRaWAN, LoRa, 3D-ray launching, fog computing, wireless sensor networks, smart irrigation systems, cloud	development of a smart irrigation system able to cover large urban areas due to the application of LP-WAN sensor nodes based on LoRa and LoRaWAN; modeling of a university campus, which includes elements like buildings, roads, green areas, or vehicles
[61]	University of Chicago/USA	IoT, Sigfox, sensors, mobile nodes, RF antenna	design and deployment of a functional wireless underground sensor network on a university campus
[62]	National Taipei University of Technology/Taiwan	IoT, wireless technologies, LoRa, sensors	sensing system to monitor and quantify the effectiveness of the green engineering projects on campus

**Table 2:** Performance of the ‘Nano’ and ‘Small’ YOLOv8 models trained during the trial run.

Parameter	‘Nano’ Model	‘Small’ Model
Layers	168	168
Parameters	3,005,843	11,125,971
Total training time [minutes]	50	83
Precision	80.9%	81.3%
Recall	91.9%	92.0%
F	86.0%	86.3%
mAP50	89.1%	90.4%
mAP75	86.9%	88.6%
mAP50-95	76.1%	77.0%

**Table 3:** The key information obtained from the IoT sensors and their impact on the project.

Information Obtained from the Collected Data	Guidelines and Conclusions for the Next Stage of Design Process
*Beam-crossing sensors:*	
Capacity of individual road sections.	Guidelines for the determination of the width of specific road sections.
Detection of two patterns of use of the space in question.	Focus on identifying who the users are in each group and how their motivations and needs change.
*CCTV cameras:*	
Determination of the actual routes used by the pedestrians moving.	Guidelines for adjusting the location of individual pedestrian routes.
Capacity of individual roads.	Guidelines for the determination of the width of specific road sections.
Selection of slow traffic locations.	Distinguishing between center-forming locations and locations where obstacles are present. *Center-forming location*: Recommendation to strengthen the center-forming function by increasing the number of seats in the specific area, adding a center-forming exhibit or service, and locating information elements there. *Presence of obstacles in the location:* Recommendation to make design changes to minimize its negative impact in the future.
Sidewalks in the central area of the WUT are blocked by cars parked there and users walk there on the roadway.	Confirmation of the proposition concerning the dysfunctional nature of space use and an argument in the discussion in favor of reducing the number of vehicles allowed in the designed area and limiting the zones that allow car traffic.

## Data Availability

The data presented in this study are available from the authors on reasonable request.
